# Oral mite anaphylaxis by *Thyreophagus entomophagus *in a child: a case report

**DOI:** 10.1186/1476-7961-7-10

**Published:** 2009-11-25

**Authors:** Javier Iglesias-Souto, Inmaculada Sánchez-Machín, Víctor Iraola, Paloma Poza, Ruperto González, Víctor Matheu

**Affiliations:** 1 Consulta de Alergia Infantil, Allergy Service, Hospital Universitario NS Candelaria, S/C Tenerife, Spain; 2 LETI, S.L., R & D Department, Madrid, Spain; 3 Unidad de Investigación, Hospital Universitario NS Candelaria, S/C Tenerife, Spain; 4 Department of Clinical Sciences-Division IV, Lund University, Sweden

## Abstract

Sensitization to *Thyreophagus entomophagus*, a storage mite, is uncommon and might produce occupational respiratory disorders in farmers. We present the first case of a child suffering anaphylaxis produced by ingestion of contaminated flour with *Thyreophagus entomophagus*.

## 

*Thyreophagus entomophagus *is a storage mite, usually sited in farms [1], but not in house dust of households [2]. Sensitization to mite species might produce occupational respiratory disorders in farmers [1,3]. However, it is unusual to live in urban houses or to produce symptoms by ingestion and there is no any report of child affected.

We encountered a 13-year-old boy suffering wheals, itching and diffuse erythema, cough and wheeze immediately after ingest a home-made *crêpe*, prepared at home with wheat flour, which was stored in kitchen for weeks. He was treated at the Emergency Department with intravenous fluids, diphenhydramine, epinephrine, and methylprednisolone, with complete symptom resolution in 2 hours. He had a previous history of mild persistent allergic rhinoconjunctivitis and sensitization to house dust mite and facial angioedema, urticaria and bronchospasm after Ibuprofen, but not any history about food allergy. Skin prick tests (SPT) to common inhalant allergens were positive to *Dermatophagoides pteronyssinus, Dermatophagoides farinae or Blomia tropicalis *and negative to the remainder inhalants and foodstuffs including wheat flour. Acoustic Rhinometry showed reversible mild obstruction. Forced spirometry showed a mild obstructive pattern with values -FVC: 3.98 (97%), FEV_1 _2.78 (79%), MEF 50% 2.22 (49%), FEF 25-75%: 1.93 (45%)-. Bronchodilator test showed a positive response with an improvement of FEV_1 _post 3.17 (+13%). After written informed consent signed by patient and his mother, open oral challenge (OOC) with different foodstuffs were performed. OOC with wheat and a commercial *crêpe *were good tolerated. Since patient's mother brought us the culprit flour, microscopic examination was performed and revealed mite contamination by *Thyreophagus entomophagus *(104 mites/gram). New SPT were done showing positive reactions with *Cheyletus spp*. and a protein extract of *Thyreophagus entomophagus *(Leti, Madrid, Spain). SPT to other storage mites were negative. Specific IgE against the extract of *Thyreophagus entomophagus *was also demonstrated *in vitro *by direct specific enzyme-immune-assay (Optical Density: 0.904; Control: 0.05) and by InmunoCAP (UniCAP, Phadia): 15,2 kU/L. Immunoblot also demonstrated IgE reactivity (figure 1).

**Figure 1:**
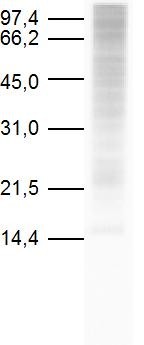
**Immunoblot of patient' serum showing IgE reactivity against the storage mite *Thyreophagus enterophagus***.

After a new written informed consent signed by patient and his mother, specific nasal provocation test was done showing positive symptoms score after instilled *Thyreophagus entomophagus *(dilution 1/10 w/v) with a drop of 30% in the minimal cross-sectional area by Acoustic Rhinometry (figure 2). Finally, after a new informed consent an open oral challenge with aspirin was done. The OOC was positive with peri-orbital angioedema.

**Figure 2:**
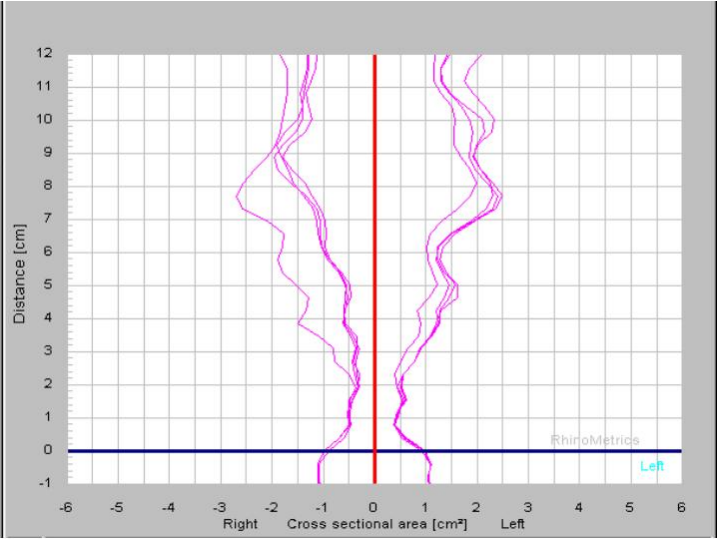
**Acoustic Rhinometry showing changes in minimal cross-sectional area after nasal provocation test with *Thyreophagus enterophagus***.

Hidden allergens [4] in allergic individuals are still a big issue [5]. Among others, hidden live organisms inside foodstuffs can provoke episodes of anaphylaxis in sensitized patients [6]. Matsumoto et al described the first case of oral mite anaphylaxis (OMA) after eating storage-mite-contaminated food by a mite [7]. Further, some other groups have reported symptoms of asthma [8] or OMA [9,10] by mite-contaminated foodstuffs. Several species of mites, such as *Dermatophagoides pteronyssinus, Dermatophagoides farinae or Blomia tropicalis *have been linked with the OMA [9,11,12], so called Pancake syndrome. However, *Thyreophagus entomophagus *has been only reported by Blanco et al [9]. This is the first report of anaphylaxis by *Thyreophagus entomophagus *in a child. Furthermore, it is the first time that a specific nasal provocation test with *Thyreophagus entomophagus *has been performed.

In our patient, the culprit foodstuff was a, previously cooked, home-made *crêpe*, This is in line of Sanchez-Borges et al, who have concluded that anaphylaxis might occur after the ingestion of heated or unheated mite-contaminated foods study [13]. In same study, authors described 28 patients with anaphylaxis triggered by ingestion of wheat-containing foodstuffs, and concluded that OMA might be more prevalent in tropical and subtropical countries than previously recognized [13].

Surprisingly, our patient had also clinical history of non-steroidal anti-inflammatory drug (NSAID) hypersensitivity, which is uncommon in children. Some authors pointed out the possible link of OMA with and NSAID hypersensitivity [9,14,15]. Furthermore, some other authors have hypothesized about a subset of individuals with a particular susceptibility for both OMA and NSAID hypersensitivity. Same authors hypothesized saying that drug hypersensitivity is coming first before than OMA called as a new triad [16].

In paediatric population, there events are more uncommon. Matsumoto and Satoh observed recently paediatric patients with OMA in Japan [17]. Wen et al described a paediatric case report of OMA in an 8-year-old Taiwanese, who was also co-sensitized to several mites including *Dermatophagoides pteronyssinus, Dermatophagoides farinae or Blomia tropicalis*. Sanchez-Borges also described a paediatric patient developed OMA [10]. As we describe above, we present the first report of anaphylaxis by *Thyreophagus entomophagus *in a child with previous sensitization to other mites. However, it is currently unknown the cross-reactivity with other mites. More and bigger studies are needed to search this possible cross-reactivity. Using fresh new flour bags could prevent these types of events in sensitized children. Mite growing should be avoided with this simple procedure of using new bags. Alternatively, previously opened bags should be transfer to plastic bags and stored inside refrigerator to avoid high humidity and temperature, optimal conditions for mite growing [3].

## Competing interests

The authors declare that they have no competing interests.

## Authors' contributions

JI-S studied the case report and wrote the initial draft of the manuscript. IS-M conceived the idea and is responsible for in vivo tests. PP was responsible for the Food Allergy Section and studied the case; VI performed *in vitro *studies. RG is responsible for the nasal study; VM analysed the data and wrote the final version of the manuscript. All authors approved the final version of the manuscript.
